# Costs of Planned Home vs. Hospital Birth in British Columbia Attended by Registered Midwives and Physicians

**DOI:** 10.1371/journal.pone.0133524

**Published:** 2015-07-17

**Authors:** Patricia A. Janssen, Craig Mitton, Jaafar Aghajanian

**Affiliations:** 1 School of Population and Public Health, University of British Columbia, Vancouver, British Columbia, Canada; 2 Child and Family Research Institute, Vancouver, British Columbia, Canada; 3 Centre for Clinical Epidemiology and Evaluation, Vancouver, British Columbia, Canada; 4 BC Centre for Epidemiologic and International Ophthalmology, Vancouver, British Columbia, Canada; Kuopio University Hospital, FINLAND

## Abstract

**Background:**

Home birth is available to women in Canada who meet eligibility requirements for low risk status after assessment by regulated midwives. While UK researchers have reported lower costs associated with planned home birth, there have been no published studies of the costs of home versus hospital birth in Canada.

**Methods:**

Costs for all women planning home birth with a regulated midwife in British Columbia, Canada were compared with those of all women who met eligibility requirements for home birth and were planning to deliver in hospital with a registered midwife, and with a sample of women of similar low risk status planning birth in the hospital with a physician. We calculated costs of physician service billings, midwifery fees, hospital in-patient costs, pharmaceuticals, home birth supplies, and transport. We compared costs among study groups using the Kruskall Wallis test for independent groups.

**Results:**

In the first 28 days postpartum, we report a $2,338 average savings per birth among women planning home birth compared to hospital birth with a midwife and $2,541 compared to hospital birth planned with a physician. In longer term outcomes, similar reductions were observed, with cost savings per birth at $1,683 compared to the planned hospital birth with a midwife, and $1,100 compared to the physician group during the first eight weeks postpartum. During the first year of life, costs for infants of mothers planning home birth were reduced overall. Cost savings compared to planned hospital births with a midwife were $810 and with a physician $1,146. Costs were similarly reduced when findings were stratified by parity.

**Conclusions:**

Planned home birth in British Columbia with a registered midwife compared to planned hospital birth is less expensive for our health care system up to 8 weeks postpartum and to one year of age for the infant.

## Introduction

Recent large population-based studies using standardized third-party ascertainment of outcomes have affirmed the safety of planned home vs. planned hospital birth attended by regulated midwives or physicians among selected women.[1–4] The desirability of the choice to give birth at home has been supported in both quantitative [5–7] and qualitative [8–12] studies. In Canada, planned home birth attended by regulated midwives was first introduced in 1994 in the province of Ontario, where the number of home births has risen to 3,000 annually.[13] In British Columbia, 11% of births are currently attended by midwives.[14] Among these, approximately 20% take place at home.[15]

The only Canadian evaluation of costs of out of hospital birth to date evaluated free standing birth centres in Quebec.[16] This study suggested that midwifery care in Quebec birth centres differs little from conventional medical care in terms of costs. An American study reported average costs in 1991 to be $1,711 for home births and for hospital births $5,382.[17] In the Netherlands, where 29% of births are planned to be at home with regulated midwives, a recent prospective study of healthy primiparous women reported a decrease in cost of 177 Euros associated with home versus hospital birth.[18] A 2008 review of economic implications of out of hospital births was unable to report definitive conclusions due to the paucity of economic literature relating to home birth, but concluded that home birth is likely to be a cost-effective option.[19] Most recently the Birthplace in England Collaborative Group, reporting on a sample of home births attended by midwives, midwifery units and in-hospital obstetric units, concluded that planned home birth was the most cost effective option for women at low risk of pregnancy complications.[20] In the current study, we present a detailed economic analysis of all home births attended by regulated midwives in the Province of British Columbia, Canada.

## Methods

The current study undertakes a government payer perspective to compare costs of planned home birth attended by regulated midwives with those of planned hospital births attended by regulated midwives and by physicians. Details of the study are presented elsewhere.[21] In brief, costs for all women planning home birth with a regulated midwife between 2001 to 2004 in British Columbia, Canada were compared with those of all women who met eligibility requirements for home birth as defined by the College of Midwives of BC [22] and were planning to deliver in hospital with a registered midwife, and with a random sample of women of similar low risk status matched on age, parity, marital status, and year of birth, and planning birth in hospital with a physician.

Linked data was obtained for this study from Population Data BC. Population Data BC is a health data resource linking heath data through a unique 10-digit personal health number assigned to all subscribers of the British Columbia Medical Services Plan (MSP). All BC residents must enroll with MSP. Personal health numbers for individuals designated to each of our study cohorts defined from the BC Perinatal Data Registry were linked to five health data registries: Medical Services Plan Payment Information;[23] and its associated Registration and Premium Billing file;[24] BC Ambulance Service;[25] Discharge Abstract Database;[26] and PharmaNet. [27]

This study was undertaken with approval from the University of British Columbia Research Ethics Board, the Children's and Women's Health Centre of British Columbia Research Review Committee, the British Columbia Ministry of Health Services, and the British Columbia College of Pharmacists.

The Medical Services Plan (MSP) Payment Information file contains data on services provided by fee-for-service practitioners to individuals covered by MSP, BC’s universal insurance program (Table 1). We assigned costs associated with fee payments to physicians by summing the amount for fee item codes specific to maternity services associated with each patient’s personal health number in the MSP records. Midwives bill the Medical Services Plan for a set fee for each proportion of a course of midwifery care, as opposed to specific maternity codes, as all of their service is related to maternity care. For our analysis of birth to 28 days postpartum, we allocated 100% of the fee schedule for labor/delivery for deliveries conducted at home by midwives. We allocated 40% if the midwife attended the birth in hospital but did not deliver the baby (after transfer of care to a physician) and 0 if the midwife was not in attendance, as per MSP protocol. We allocated two thirds of the cost of a course of postpartum care for 28 days if the midwife billed for postpartum care. For our analysis of birth to 56 days (8 weeks) we similarly assigned costs of labour/delivery and allocated 100% of the postpartum component of care. Tota costs from which proportionate costs were derived were obtained from the Medical Services Payment Plan.[28]

**Table 1:** Data Sources.

Data Source	Data
BC Perinatal Data Registry	Personal Health Numbers; maternal and newborn characteristics, pregnancy and birth information.
Medical Services Plan Payment Information	Services provided by fee-for-service practitioners to individuals covered by British Columbia’s Medical Services Plan, and codes for service fees.
Registration and Premium Billing file	Information on Medical Services Plan registration.
BC Ambulance Service	Mode of emergency transport, air or ground, and cost estimates for each.
Discharge Abstract Database	Case Mix Groups and Resource Intensity Weights for each individual who was discharged or transferred from an acute care hospital in BC.
PharmaNet	The ingredient cost of drugs, professional fees, and third-party costs (administrative costs of adjudicators/employers).

Mode of emergency transport, air or ground, was obtained from BC Ambulance Service data files obtained by Population Data BC. In consultation with BC Ambulance Service, (personal communication D. Andrusiek, Research Director, Medical Programs, Emergency and Health Services Commission of BC Ambulance Service), ambulance costs were attributed as follows: $500 for ground, $1000 for air, and $1500 for a combination of air and ground. If transport included both mother and baby, the cost is listed for the infant only.

The Discharge Abstract Database houses data on discharges, transfers and deaths of in-patients and day surgery patients from acute care hospitals in BC. The hospitalization cost was derived by multiplying the In-Patient Resource Intensity Weight (P-RIW) by the Cost Per Weighted Case (CPWC) for the corresponding site and fiscal year. The RIW measures the intensity of resources used based on patient diagnosis, surgical procedure performed and the case mix group assigned to the individual patient. Case mix is an inpatient grouping methodology used in Canada to create discrete clusters of patients using clinical, administrative and resource consumption data. The case mix group takes into consideration the patient’s age, health status, and discharge status. The result is groups of patients that are clinically similar and/or homogeneous with respect to hospital resources used.[29] The Canadian Institute for Health Information (CIHI) defines RIWs for case mix groups. RIWs for individuals are available from the Discharge Abstract Database. To convert the RIWs into actual dollars, the RIW is multiplied by the CPWC. When the total expenditures for inpatient care in a particular acute care hospital for one year is divided by the total weighted cases of the same hospital during the same year, the result is the average cost of providing care to a patient with a weighted case value of 1.00. Thus, the CPWC is the cost of a stay with a weight of 1.00. The CPWC is different for each hospital each year. Province-wide CPWCs for the study period were obtained from the BC Ministry of Health. We applied the CPWC value specific to each year and each hospital when computing costs.[30]

PharmaNet records all prescriptions dispensed by British Columbia pharmacists in an outpatient setting. PharmaNet is administered by the British Columbia Ministry of Health for residents of BC. PharmaNet records include the ingredient cost, professional fees, and third-party costs (administrative costs of adjudicators/employers). Records also include non-drug items such as diabetic test strips. Cost data was not available for residents paid through federal plans, including federal police officers, veterans or individuals funded through Indian Affairs, but ‘quantity dispensed’ and ‘days supply’ was available and corresponding costs were estimated from provincial costs for these entities.

To capture costs associated with the intrapartum period, we included costs from all data sources for the 48 hour period before the date and time of birth for the mother. In our first analysis, we included maternal costs until and including 28 days postpartum to correspond to the completion of the neonatal period for the baby (Mom -2 to +28, Baby +28). Our second framework for comparison was the postpartum period for the mother, defined as 8 weeks or 56 days, and the first year of life or 365 days for the infant (Mom -2 to +56, Baby +365). To exclude births by non-BC residents, all mothers included had to be registered with the provincial medical services plan during the follow-up period.

We excluded from our analysis, 217 mother-baby pairs in which the babies had one or more ICD-10 codes for congenital malformations. After removal of one birth with data entry errors, and 19 births in hospitals where the cost per weighted case (CPWC) was not available, the study population comprised records related to 9,864 live births.

We compared costs among study groups using the non-parametric Kruskal Wallis test for independent samples, as distributions were not normally distributed. We report mean costs per group, and groups stratified by parity. The data were analysed using SAS software (version 9.3, SAS Institute, Cary, N.C.)

## Results

In total our study sample included 9864 women: all women who planned a home birth with a regulated midwife (n = 2243); all planned hospital midwife-attended births meeting the eligibility requirements for home birth (n = 3610) and a sample of women planning hospital birth with a physician, also meeting eligibility requirements for home birth and matched on age category, parity, and restricted to hospitals in which midwives had admitting privileges (n = 4011). Comparison groups were similar with respect to age, lone parent status, income quintile, BMI, use of substances and gestational age at start of prenatal care (Table 2).

**Table 2:** Sociodemographic and Pregnancy Related Characteristics of Study Participants.

	Planned Home Birth with Midwife	Planned Hospital Birth with Midwife	Planned Hospital Birth with Physician
	n = 2,243	n = 3,610	n = 4,011
	n (%)	n (%)	n (%)
Age (years)			
15–19	30 (1.3)	79 (2.2)	64 (1.6)
20–24	263 (11.7)	438 (12.1)	477 (11.9)
25–29	690 (30.8)	1068 (29.6)	1215 (30.3)
30–34	792 (35.3)	1279 (35.4)	1437 (35.8)
≥ 35	468 (20.9)	746 (20.7)	818 (20.4)
Lone parent			
yes	67 (3.1)	165 (4.8)	105 (2.7)
no	2100 (96.9)	3265 (95.2)	3773 (97.3)
missing	76	180	133
Income Quintile			
1	505 (23.5)	693 (19.9)	786 (20.2)
2	463 (21.6)	673 (19.3)	876 (22.5)
3	396 (18.4)	681 (19.5)	770 (19.8)
4	424 (19.7)	766 (22.0)	772 (19.8)
5	360 (16.8)	669 (19.2)	686 (17.6)
Missing	95	128	121
Body Mass Index, mean (sd)	22.6 (4.0)	23.3 (4.3)	23.2 (4.4)
Alcohol	3 (0.1)	16 (0.4)	21 (0.5)
Illicit drug use	25 (1.1)	45 (1.2)	48 (1.2)
Smoking status			
Current	116 (5.2)	267 (7.4)	346 (8.5)
Former	198 (8.8)	327 (9.1)	168 (4.2)
None	1929 (86.0)	3016 (83.5)	3497 (87.2)
Nulliparous	926 (41.3)	1797 (49.8)	1619 (40.4)
Mean gestational age at 1^st^ prenatal visit (wks), mean (sd)	11.9 (6.7)	11.8 (6.7)	11.4 (5.3)

During the initial 28 days postpartum, average costs per mother were significantly reduced among women planning home birth compared to hospital birth, planned either with a midwife or a physician (Table 3). Compared to those who planned hospital birth with a midwife, provider fees, hospital charges, and pharmaceutical costs were significantly less. Compared to planned hospital births with a physician, provider fees and transport costs were higher and hospital costs and pharmaceutical costs were less. Similarly, average costs per newborn during this period, corresponding to the early neonatal period, were significantly reduced in the home birth group. Provider fees and pharmaceutical costs were lower in the home birth group compared to both planned hospital birth groups. Our data indicate a $2,338 average cost savings per birth among women planning home birth compared to hospital birth with a midwife and $2,541 compared to hospital birth planned with a physician.

**Table 3:** Average Costs of Planned Home vs. Hospital Birth by Regulated Midwife or Physician, 0–28 days.

	Planned Home Birth with Midwife	Planned Hospital Birth with Midwife	p-value	Planned Hospital Birth with Physician	p-value
	n = 2,243	n = 3,610		n = 4,011	
**Maternal**					
Physician fee (Dollars Cdn)	183	347	<.0001	965	<.0001
Midwifery fee	922	906	<.0001	0	<.0001
Hospital charges	684	2,276	<.0001	2,524	<.0001
Transport	13	7	0.001	5	.0003
Pharmaceuticals	6	10	<.0001	13	<.0001
Home birth supplies	50	0		0	
**Total**	**1,858**	**3,546**	<.0001	**3,507**	<.0001
**Newborn**					
Physician fee	70	130	<.0001	266	<.0001
Hospital charges	343	932	<.0001	1,040	<.0001
Transport	4	5	0.68	2	0.014
Pharmaceuticals	0	1	0.001	2	<.0001
**Total**	**417**	**1,067**	<.0001	**1,310**	<.0001
**Total Maternal/Newborn**	**2,275**	**4,613**	<.0001	**4,816**	<.0001

Our findings were similar among nulliparous women, for whom overall average costs among women planning home birth were significantly reduced by $2,122 and $2,518 for planned hospital birth with a midwife and physician respectively (Table 4). Among multiparous women, corresponding cost savings were $2,307 and $2,579 per birth (Table 4).

**Table 4:** Average Costs of Planned Home vs. Hospital Birth by Regulated Midwife or Physician and Parity, 0–28 days.

	Planned Home Birth with Midwife	Planned Hospital Birth with Midwife	p-value	Planned Hospital Birth with Physician	p-value
**Nulliparous Women**	**n = 926**	**n = 1797**		**n = 1619**	
**Maternal**					
Physician fee (Dollars Cdn)	318	551	<.0001	1,196	<.0001
Midwifery fee	877	858	<.0001	0	<.0001
Hospital charges	1,187	2,574	<.0001	2,924	<.0001
Transport	17	8	0.006	6	0.0003
Pharmaceuticals	7	11	0.0001	16	<.0001
Home birth supplies	50	0	<.0001	0	<.0001
**Total**	**2,456**	**4,002**		**4,142**	
**Newborn**					
Physician fee	102	166	0.0001	307	<.0001
Hospital charges	558	1069	<.0001	1,185	<.0001
Transport	4	5	0.93	4	0.54
Pharmaceuticals	0.4	0.8	0.002	2	<.0001
**Total**	**665**	**1,241**		**1,498**	
**Total Maternal/Newborn**	**3,121**	**5,243**	<.0001	**5,639**	<.0001
**Multiparous Women**	**n = 1,317**	**n = 1,813**		**n = 2,392**	
**Maternal**					
Physician fee (Dollars Cdn)	88	144	0.0002	808	<.0001
Midwifery fee	954	954	<.0001	0	<.0001
Hospital charges	330	1,981	<.0001	2,253	<.0001
Transport	10	6	0.03	4	0.0031
Pharmaceuticals	5	9	0.001	12	<.0001
Home birth supplies	50	0		0	
**Total**	**1,438**	**3,093**	<.0001	**3,077**	<.0001
**Newborn**					
Physician fee	47	94	<.0001	238	<.0001
Hospital charges	191	795	<.0001	942	<.0001
Transport	5	5	0.68	2	0.006
Pharmaceuticals	0.5	1	0.0001	2	<.0001
**Total**	**243**	**895**	<.0001	**1,183**	<.0001
**Total Maternal/Newborn**	**1,681**	**3,988**	<.0001	**4,260**	<.0001

During the first 56 days, corresponding to the eight week postpartum period, maternal costs for women planning home birth were significantly reduced overall and for sub-categories of hospital and pharmaceutical costs. Costs for transport were higher in the home birth group. Costs savings per birth were $1,683 compared to the planned hospital birth with a midwife, and $1,100 compared to the physician group (Table 5).

**Table 5:** Costs of Planned Home vs. Hospital Birth by Regulated Midwife or Physician, 0–56 days (Maternal) and 0–1 Year (Infant).

	Planned Home Birth with Midwife	Planned Hospital Birth With Midwife	p-value	Planned Hospital Birth with Physician	p-value
	n = 2243	n = 3610		n = 4011	
**Maternal**					
Physician fee	205	379	<.0001	1,007	<.0001
Midwifery fee	1,500	1,459	<.0001	0	<.0001
Hospital charges	689	2,289	<.0001	2,531	<.0001
Transport	13	7	0.0016	5	0.0001
Pharmaceuticals	11	18	<.0001	26	0.0001
Home birth supplies	50	0		0	
**Total**	**2,469**	**4,152**	<.0001	**3,569**	<.0001
**Newborn**					
Physician fee	288	445	<.0001	649	<.0001
Hospital charges	540	1,177	<.0001	1,294	<.0001
Transport	9	10	0.38	7	0.15
Pharmaceuticals	17	32	<.0001	50	<.0001
**Total**	**854**	**1,664**	<.0001	**2,000**	<.0001

For nulliparous women these cost savings were $1,514 and $1,133 and for multiparous women $1,679 and $1,095. (Table 6).

**Table 6:** Costs of Planned Home vs. Hospital Birth by Regulated Midwife or Physician, 0–56 days (Maternal) and 0–1 Year (Infant) by Parity.

	Planned Home Birth with Midwife	Planned Hospital Birth With Midwife	p-value	Planned Hospital Birth with Physician	p-value
**Nulliparous Women**	**n = 926**	**n = 1797**		**n = 1619**	
**Maternal**					
Physician fee	345	589	<.0001	1,243	<.0001
Midwifery fee	1,455	1,379	<.0001	0	<.0001
Hospital charges	1,193	2,587	<.0001	2,927	<.0001
Transport	17	8	0.07	6	0.0003
Pharmaceuticals	13	22	<.0001	29	0.0001
Home birth supplies	50	0		0	
**Total**	**3,072**	**4,586**	<.0001	**4,205**	<.0001
**Newborn**					
Physician fee	334	502	<.0001	712	<.0001
Hospital charges	740	1,301	0.01	1,409	0.0002
Transport	6	8	0.49	8	0.94
Pharmaceuticals	13	33	<.0001	61	0.0001
**Total**	**1,094**	**1,843**	<.0001	**2,189**	<.0001
**Multiparous Women**	**n = 1,317**	**n = 1,813**		**n = 2,302**	
**Maternal**					
Physician fee	107	171	<.0001	848	<.0001
Midwifery fee	1,533	1,538	<.0001	0	<.0001
Hospital charges	335	1,993	<.0001	2,263	<.0001
Transport	10	6	0.05	4	0.005
Pharmaceuticals	10	15	0.0001	24	0.0001
Home birth supplies	50	0		0	
**Total**	**2,044**	**3,723**	<.0001	**3,139**	<.0001
**Newborn**					
Physician fee	257	389	<.0001	606	<.0001
Hospital charges	398	1,055	<.0001	1,216	<.0001
Transport	11	12	0.45	6	0.06
Pharmaceuticals	19	31	<.0001	43	<.0001
**Total**	**685**	**1,487**	<.0001	**1,871**	<.0001

During the first year of life, costs for infants of mothers planning home birth were reduced overall, and for physician fees and pharmaceutical costs compared to both planned hospital cohorts. Cost savings compared to planned hospital births with a midwife were $810 and with a physician $1,146 (Table 5).

Costs were similarly reduced when findings were stratified by parity. Cost savings among infants of primiparous women were $749 and $1,095 for hospital births planned with a midwife and physician respectively and for infants of multiparous women were $802 and $1,186 (Table 6).

## Discussion

Our study demonstrates a significant cost savings for planned home birth in British Columbia with a regulated midwife compared to planned hospital birth, either with a regulated midwife or with a physician. As expected, transport costs were higher for mothers in the home birth group, but provider fees, hospital costs and pharmaceutical costs were lower for both mothers and infants. We would expect hospital costs to be lower since most women in the planned home birth would not be admitted to hospital. Provider fees and pharmaceutical costs are reduced in both midwifery cohorts compared to the physician group and likely reflect both the reduced rates of interventions among women receiving care by midwives, and, since these differences persist beyond 28 days, self-selection to planned home birth of women who are particularly healthy and do not wish to have pharmacological interventions during labour and birth. This is the first study of home birth to extend analysis of cost to the conclusion of the postpartum period for mothers and to one year for infants. This is noteworthy because “hidden” risks of home birth, that is morbidity manifesting beyond the immediate postpartum period, if it existed, would be reflected in higher costs during the prolonged period of observation in this study. However, delayed morbidity does not appear to be a consequence of home birth.

Due to differences in how costs are assigned across studies and countries, our findings may be cautiously compared to those of Hendrix et al. in the Netherlands, comparing costs of births to nulliparous women planning birth at home with a regulated midwife versus women planning birth in short stay units attended by either midwives or family practice physicians.[18] The study examined 100 midwifery practices sampled at random from across the Netherlands. Costs for provider fees, hospital, and transport from the intrapartum period to six weeks postpartum were €339 ($495 Cdn) less in the planned home birth group. This analysis, however, does not separate births attended by midwives and physicians.

A cost analysis of home birth from Washington State, USA during the same time period as ours, reported cost savings of $2,971 for planned home births attended by licensed midwives vs. planned hospital births resulting in vaginal delivery attended by midwives and $5, 550 for hospital births attended by midwives resulting in cesarean delivery. These differences, which correspond only to the intrapartum period, are larger than ours, but again must be viewed cautiously as costs in a Canadian context do not necessarily align well with costs (or charges) reported in US studies.[31]

A British study reporting on 142 of 147 regional health authorities (“trusts”) estimated costs for provider fees and salaries, hospital, transport and pharmaceutical costs from finance departments of participating trusts, consultations with midwives, and national sources of data. The mean savings for home births (maternal and newborn) during the intrapartum period versus obstetrical units was £564.6 ($1073.93 Cdn).

Strengths of our study include complete ascertainment of planned home and of hospital births attended by midwives in an entire province. The same midwives attend both home and hospital birth in British Columbia since they are required to offer eligible women the choice of either setting. Our comparison of home vs. hospital in the midwifery groups therefore reflects a true comparison of place of birth un-confounded by type of caregiver. Both midwifery groups and our random sample of physician-attended birth were of comparable low risk status. We report costs assigned to individual hospitals within each study year, accounting for potential confounding by hospital size and location. In addition, our period of observation was longer than reported in the literature to date. Our study is limited by our inability to ascertain actual transport costs, although the proportion of costs for transportation was less than one percent of all costs. More importantly, our hospital costing data is limited to membership in case mix groups rather than individual costs as individual costing data is not collected by hospitals in BC. The number of case mix groups that can be assigned to mothers is 25 and to infants 31, however, indicating a wide range of designations for complexity. We were also unable to include costs incurred due to lost productivity, although it might reasonably be expected that differences between our groups would not be observed. In addition, our data do not include costs from long term morbidity such as neurological sequelae that may not have manifested in the first year of life.

## Conclusion

We conclude that planned home birth in British Columbia with a registered midwife compared to birth planned for hospital with a registered midwife or a physician is less expensive for our health care system for mothers up to 8 weeks postpartum and infants up to one year of age. Our findings should reassure health planners and policy makers that there are not deferred excess costs associated with planned home birth with a registered midwife and encourage home birth in similar settings as a choice for healthy women.

## Supporting Information

S1 Fig Janssen Home vs Hospital Birth Outcomes paper. (PDF)
