# Supplementary material for: Costs of Planned Home vs. Hospital Birth in British Columbia Attended by Registered Midwives and Physicians
Source: PLoS One. 2015 Jul 17;10(7):e0133524. doi: 10.1371/journal.pone.0133524 (PMC4505882; doi:10.1371/journal.pone.0133524)
Supplement: S1 Fig — (PDF) [file pone.0133524.s001.pdf]

CMAJ

Research

Outcomes of planned home birth with registered midwife versus planned hospital birth with midwife or physician

Patricia A. Janssen PhD, Lee Saxell MA, Lesley A. Page PhD, Michael C. Klein MD, Robert M. Liston MD, Shoo K. Lee MBBS PhD

Previously published at www.cmaj.ca

See related commentary by McLachlan and Forster, page 359

Abstract

Background: Studies of planned home births attended by registered midwives have been limited by incomplete data, nonrepresentative sampling, inadequate statistical power and the inability to exclude unplanned home births. We compared the outcomes of planned home births attended by midwives with those of planned hospital births attended by midwives or physicians.

Methods: We included all planned home births attended by registered midwives from Jan. 1, 2000, to Dec. 31, 2004, in British Columbia, Canada (n = 2889), and all planned hospital births meeting the eligibility requirements for home birth that were attended by the same cohort of midwives (n = 4752). We also included a matched sample of physician-attended planned hospital births (n = 5331). The primary outcome measure was perinatal mortality; secondary outcomes were obstetric interventions and adverse maternal and neonatal outcomes.

Results: The rate of perinatal death per 1000 births was 0.35 (95% confidence interval [CI] 0.00–1.03) in the group of planned home births; the rate in the group of planned hospital births was 0.57 (95% CI 0.00–1.43) among women attended by a midwife and 0.64 (95% CI 0.00–1.56) among those attended by a physician. Women in the planned home-birth group were significantly less likely than those who planned a midwife-attended hospital birth to have obstetric interventions (e.g., electronic fetal monitoring, relative risk [RR] 0.32, 95% CI 0.29–0.36; assisted vaginal delivery, RR 0.41, 95% 0.33–0.52) or adverse maternal outcomes (e.g., third- or fourth-degree perineal tear, RR 0.41, 95% CI 0.28–0.59; postpartum hemorrhage, RR 0.62, 95% CI 0.49–0.77). The findings were similar in the comparison with physician-assisted hospital births. Newborns in the home-birth group were less likely than those in the midwife-attended hospital-birth group to require resuscitation at birth (RR 0.23, 95% CI 0.14–0.37) or oxygen therapy beyond 24 hours (RR 0.37, 95% CI 0.24–0.59). The findings were similar in the comparison with newborns in the physician-assisted hospital births; in addition, newborns in the home-birth group were less likely to have meconium aspiration (RR 0.45, 95% CI 0.21–0.93) and more likely to be admitted to hospital or readmitted if born in hospital (RR 1.39, 95% CI 1.09–1.85).

Interpretation: Planned home birth attended by a registered midwife was associated with very low and comparable rates of perinatal death and reduced rates of obstetric interventions and other adverse perinatal outcomes compared with planned hospital birth attended by a midwife or physician.

Une version française de ce résumé est disponible à l'adresse www.cmaj.ca/cgi/content/full/cmaj.081869/DC1

The debate about the safety of home births continues in the literature, professional policy and practice. Planned home births attended by registered professional attendants have not been associated with an increased risk of adverse perinatal outcomes in large studies in North America,[1–3] the United Kingdom,[4] Europe,[5–8] Australia[9] and New Zealand.[10] However, these studies have been limited by the voluntary submission of data,[1,4,5,8,10] nonrepresentative sampling,[6,7] lack of appropriate comparison groups,[1,7,9] inadequate statistical power[3,8] and the inability to exclude unplanned home births from the study sample.[2,11,12]

The Society of Obstetricians and Gynaecologists of Canada encourages research into the safety of all birth settings. It does not take a specific stand on home birth.[13] In 2008, the American College of Obstetricians and Gynecologists reiterated its long-standing opposition to home births, stating that the choice to deliver at home places the process of giving birth ahead of the goal of having a healthy baby.[14] In contrast, the Royal College of Obstetricians and Gynaecologists in the United Kingdom

From the School of Population and Public Health (Janssen), the Departments of Family Practice (Klein) and Obstetrics and Gynecology (Janssen, Liston) and the Division of Midwifery (Saxell), Faculty of Medicine, University of British Columbia, Vancouver, BC; the Child and Family Research Institute (Janssen, Klein, Liston), Vancouver, BC; the Nightingale School of Nursing and Midwifery (Page), King's College, London, UK; the Department of Pediatrics (Lee); and the Integrated Centre for Care Advancement Through Research (Lee), University of Alberta, Edmonton, Alta.

Cite as *CMAJ* 2009. DOI:10.1503/cmaj.081869

DOI:10.1503/cmaj.081869

© 2009 Canadian Medical Association or its licensors

has issued a statement supporting home birth as a viable choice for women with uncomplicated pregnancies.[15]

In this study, we ascertained outcomes of all planned home births attended by registered midwives in an entire health region with a single-payer universal health care system. We compared them with the outcomes of all planned hospital births that met the criteria for home birth and were attended by the same cohort of midwives. We also compared the outcomes of a matched sample of women of similar risk status who planned to deliver in hospital with a physician in attendance.

Methods

Setting

The study was conducted in British Columbia, a province in Canada with a population of more than 4.4 million. Midwives are registered by the College of Midwives of British Columbia if they have a baccalaureate degree in midwifery from a Canadian university. If they trained outside of Canada, they are registered by the college after passing written, oral and practice-based exams. Registered midwives are mandated to offer women the choice to deliver in hospital or at home if they meet the eligibility criteria for home birth defined by the college (Box 1). Midwifery care is funded by the provincial Ministry of Health and is accessible to all women in the province who meet the standards for low obstetric risk (Box 1).

Box 1: Eligibility requirements for home birth mandated by the College of Midwives of British Columbia

- Absence of significant pre-existing disease, including heart disease, hypertensive chronic renal disease or type 1 diabetes
- Absence of significant disease arising during pregnancy, including pregnancy-induced hypertension with proteinuria (> 0.3 g/L by urine dipstick), antepartum hemorrhage after 20 weeks' gestation, gestational diabetes requiring insulin, active genital herpes, placenta previa or placental abruption
- Singleton fetus
- Cephalic presentation
- Gestational age greater than 36 and less than 41 completed weeks of pregnancy
- Mother has had no more than 1 previous cesarean section
- Labour is spontaneous or induced on an outpatient basis
- Mother has not been transferred to the delivery hospital from a referring hospital

Study population

In the study group, we included all births in British Columbia between Jan. 1, 2000, and Dec. 31, 2004, that were planned to take place at the woman's home at the onset of labour. Membership in the study group was ascertained in part from the provincial Perinatal Database Registry, which captures all births in the province and is cross-referenced with the Department of Vital Statistics. Maternity care is documented on standardized forms issued by the province's Perinatal Health Program. The eligibility requirements for home birth mandated by the provincial College of Midwives are provided in Box 1.

Because our goal was to better inform childbearing women and their caregivers of the potential consequences of home birth, we chose to study the planned rather than the actual place of birth. The planned place of birth at the onset of labour is documented for every birth on rosters submitted to the College of Midwives by the primary midwife at 8 weeks postpartum. This information is matched to registry data by use of unique personal health numbers.

We did not exclude planned home births during which the fetal presentation was determined to be breech after the onset of labour. We also did not exclude women who had had 1 previous cesarean birth, because these women are eligible for home birth under current standards of practice.[16] Vaginal birth after a cesarean section is known to carry additional risk to mother and newborn.[17] Accordingly, our comparison groups did not include women who had had a prior cesarean birth. In a subgroup analysis, we restricted the home-birth group to women who had no prior cesarean delivery.

In a second subgroup analysis, we included only women whose labour was spontaneous. We did this to exclude women who may have had a home birth after a successful outpatient induction of labour with intravaginal prostaglandins or amniotomy.

We had 2 comparison groups of planned hospital births. The first comprised all births during the study period to women who planned to give birth in hospital with a registered midwife in attendance. We selected births for this group from the Perinatal Database Registry if a midwife was in attendance during labour and the rosters of the College of Midwives indicated that the birth was planned to be in hospital. We further restricted the group to women who met the eligibility criteria for home birth. The midwives who conducted hospital births were the same cohort of midwives who conducted home births. This group, therefore, allows for comparison of birth outcomes attributable to planned place of birth unconfounded by type of caregiver.

Our second comparison group comprised all births during the study group to women who planned to give birth in hospital with a physician in attendance. Given that midwives attend only 6% of births in British Columbia, the majority of women who choose hospital birth plan to have a physician attendant.[18] There are no physician-attended home births, because such attendance is outside the scope of practice of physicians. We matched physician-attended births that met the eligibility criteria for home birth individually to each home birth on a 2:1 ratio. Parameters were year of birth, parity (primiparous v. multiparous), single parent (yes v. no), maternal age (< 15, 15–19, 20–24, 25–29, 30–34 or > 35 years) and the hospital where the midwife conducting the index home birth had hospital privileges. To control as much as possible for variables such as urban versus rural setting, size of hospital and predominance of ethnic groups, we restricted physician-attended births to those in hospitals where midwives held privileges. For each home birth, we randomly selected a comparison case from the eligible matches.

For all women included in the study, we collected data on their age, height, weight before pregnancy, body mass index,

income quintile, drug and alcohol use (v. no use) during pregnancy, smoking status, status of parenthood (single v. other), parity, gestational age at first prenatal visit, number of antenatal visits and history of ultrasonography before 20 weeks' gestation. For income quintiles, we used average household incomes, adjusted for size of household, within a given area of census enumeration derived from postal codes.

Outcome measures

Our primary outcome measure was the rate of perinatal death, defined as stillbirth after 20 weeks' gestation or death in the first 7 days of life. We projected 2750 home births for analysis over the study period and therefore planned to have 92% power to estimate perinatal death rates within 3 births per 1000 with 95% confidence.[19]

Our secondary outcome measures were obstetric interventions and adverse neonatal and maternal outcomes. We derived neonatal outcomes using data obtained from the Perinatal Database Registry. Validation studies have recorded accuracy rates of 97% over all data fields for this database.[20] The rate of missing data is less than 0.01%.[20] The registry links outcomes for infants transferred from a birth hospital to referring hospitals up to the final discharge home or to 1 year of age, whichever is shorter. Linked outcomes for newborns readmitted to any hospital up to 28 days of age are included. In addition, the registry contains standard procedural and diagnostic codes of the *International Statistical Classification of Diseases and Related Health Problems*, 10th revision (ICD-10), abstracted from patient records after discharge.

Our study was approved by the University of British Columbia Clinical Ethics Research Board.

Statistical analysis

We calculated relative risks for outcomes analyzed within cohorts related to the planned birth setting and caregiver and not where the birth actually occurred. We weighted relative risks when adjustment altered the summary relative risk by at least 10%.[21]

Results

During the 5-year study period, 2899 women attended by a registered midwife began labour with the intention of giving birth at home; 4752 who met the eligibility criteria for planned home birth began labour with the intention of giving birth in hospital. Our physician-attended cohort comprised 5331 women. We excluded women who required oxytocin for induction of labour after the 2:1 matching with our study group, because we learned during the study that the policy with respect to the use of oxytocin for induction of labour was outside the scope of practice for midwives and family physicians in some hospitals.

Compared with women who planned a midwife-attended hospital birth, those who planned a home birth were less likely to be single parents or to be nulliparous (Table 1).

Perinatal mortality

The rate of perinatal death per 1000 births was very low and comparable in all 3 groups: it was 0.35 (95% confidence interval [CI] 0.00–1.03) among the planned home births, 0.57 (95% CI 0.00–1.43) among the planned hospital births attended by a midwife and 0.64 (95% CI 0.00–1.56) among the planned hospital births attended by a physician. There were no deaths between 8 and 28 days of life.

Obstetric interventions

Of the women who planned to give birth at home, 2285 (78.8%) did so. Of those who planned a hospital birth with a midwife in attendance, 4604 (96.9%) did so.

The frequency of obstetric interventions and their indications are listed in Table 2. Compared with women who planned a hospital birth with a midwife or physician in attendance, those who planned a home birth were significantly less likely to experience any of the obstetric interventions we assessed, including electronic fetal monitoring, augmentation of labour, assisted vaginal delivery, cesarean delivery and episiotomy (Table 3).

Adverse maternal and neonatal outcomes

Adverse maternal outcomes were rare in all 3 groups (Table 2). Compared with women who planned a hospital birth with a midwife in attendance, those who planned a home birth were significantly less likely to have a third- or fourth-degree perineal tear (adjusted relative risk [RR] 0.43, 95% CI 0.29–0.63), postpartum hemorrhage (RR 0.62, 95% CI 0.49–0.77) or pyrexia (RR 0.45, 95% CI 0.29–0.76) (Table 3). The rate of infection overall, although lower in the home-birth group, did not differ significantly between these 2 groups (RR 0.39 (0.13–1.14). The risk of all adverse maternal outcomes assessed was significantly lower among the women who planned a home birth than among those who planned a physician-attended hospital birth (Table 3).

Compared with women who planned a midwife-attended hospital birth, those who planned a home birth were less likely to have a newborn who had birth trauma (RR 0.26, 95% CI 0.11–0.58), required resuscitation at birth (RR 0.23, 95% CI 0.14–0.37) or required oxygen therapy beyond 24 hours (RR 0.37, 95% CI 0.24–0.59) (Table 4; see also Appendix 1, available at www.cmaj.ca/cgi/content/full/cmaj.081869/DC2).

When compared with newborns of women who planned a hospital birth attended by a physician, those whose mothers planned a home birth were similarly at reduced risk of birth trauma (RR 0.33, 95% CI 0.15–0.74), resuscitation at birth (RR 0.56, 95% CI 0.32–0.96) and oxygen therapy behond 24 hours (RR 0.38, 95% CI 0.24–0.61) (Table 4, Appendix 1). In addition, they were less likely to have meconium aspiration (RR 0.45, 95% CI 0.21–0.93) and more likely to be admitted to hospital or readmitted if born in hospital (RR 1.39, 95% CI 1.09–1.85).

We observed no significant differences between the home-birth group and either comparison group with respect to a 5-minute Apgar score of less than 7, a diagnosis of asphyxia at birth, seizures, or the need for assisted ventilation beyond the first 24 hours of life.

When we excluded the 88 women who had a previous cesarean delivery from the home-birth cohort in the subgroup analysis, the relative risks of obstetric interventions and adverse maternal and neonatal outcomes did not change

substantively and did not alter any of our conclusions. In the subgroup analysis in which we excluded women whose labour was induced by outpatient administration of prostaglandins, amniotomy or both (118 [4.1%] of women in the home-birth group, 344 [7.2%] of those who planned a midwife-attended hospital birth and 778 [14.6%] of those who planned a physician-attended hospital birth), the relative risks of obstetric interventions and adverse maternal and neonatal outcomes did not change significantly. When we restricted the home-birth group to women who actually gave birth at home, the rates of adverse maternal and newborn outcomes did not differ significantly from those among all planned home births. There were no perinatal deaths among births that took place at home.

Interpretation

The decision to plan a birth attended by a registered midwife at home versus in hospital was associated with very low and comparable rates of perinatal death. Women who planned a home birth were at reduced risk of all obstetric interventions assessed and were at similar or reduced risk of adverse maternal outcomes compared with women who planned to give birth in hospital accompanied by a midwife or physician. Newborns whose mothers planned a home birth were at similar or reduced risk of fetal and neonatal morbidity compared with newborns whose mothers planned a hospital birth, except for admission to hospital (or readmission if born in hospital), which was more likely compared with newborns

Table 1: Characteristics of 12 982 women in British Columbia who planned a home birth or hospital birth attended by a registered midwife or planned a hospital birth attended by a physician during 2000–2004

Characteristic	Group; no. (%) of women*		
	Planned home birth with midwife *n* = 2899	Planned hospital birth with midwife *n* = 4752	Planned hospital birth with physician *n* = 5331
Age, yr			
15–19	48 (1.7)	116 (2.4)	92 (1.7)
20–24	336 (11.6)	584 (12.3)	629 (11.8)
25–29	892 (30.8)	1371 (28.9)	1644 (30.8)
30–34	1025 (35.4)	1682 (35.4)	1883 (35.3)
≥ 35	598 (20.6)	999 (21.0)	1083 (20.3)
Single parent	91 (3.1)	252 (5.3)	163 (3.1)
Income quintile†			
1 (lowest)	650 (23.4)	906 (19.8)	1088 (21.1)
2	593 (21.3)	910 (19.9)	1163 (22.6)
3	525 (18.9)	913 (20.0)	1006 (19.5)
4	543 (19.5)	984 (21.5)	1020 (19.8)
5 (highest)	460 (16.5)	862 (18.8)	875 (17.0)
Height, cm, mean (SD)	166.5 (6.6)	166.4 (7.0)	164.3 (7.0)
Weight before pregnancy, kg, mean (SD)	63.1 (11.7)	64.4 (12.7)	62.6 (13.0)
Body mass index, mean (SD)	22.8 (4.0)	23.3 (4.3)	23.2 (4.3)
Use of illicit drugs during pregnancy	39 (1.3)	57 (1.2)	71 (1.3)
Use of alcohol during pregnancy	10 (0.3)	25 (0.5)	35 (0.7)
Smoking status			
Current	166 (5.7)	375 (7.9)	487 (9.1)
Former	256 (8.8)	417 (8.8)	211 (4.0)
Never	2477 (85.4)	3960 (83.3)	4633 (86.9)
Nulliparous	1215 (41.9)	2428 (51.1)	2204 (41.3)
Gestational age at first prenatal contact, wk, mean (SD)	12.2 (7.0)	12.2 (6.8)	11.8 (5.9)
No. of antenatal visits, mean (SD)	11.8 (3.3)	11.2 (3.6)	9.3 (2.7)
Ultrasound < 20 wk gestation	1707 (58.9)	3371 (70.9)	4027 (75.5)

Note: SD = standard deviation.
*Unless indicated otherwise.
†Percentages are based on the number of people for whom income data were available (2781 in the planned home-birth group, 4575 in the planned midwife-attended hospital-birth group and 5152 in the planned physician-attended hospital-birth group).

Table 2: Obstetric interventions and maternal outcomes among the 12 982 women in the study

Variable	Group; no. (%) of women		
	Planned home birth with midwife *n* = 2899	Planned hospital birth with midwife *n* = 4752	Planned hospital birth with physician *n* = 5331
**Obstetric intervention**			
Electronic fetal monitoring	394 (13.6)	1992 (41.9)	4201 (78.8)
External tocometer	389 (13.4)	1970 (41.5)	4164 (78.1)
Fetal scalp electrode	60 (2.1)	247 (5.2)	548 (10.3)
Augmentation of labour	688 (23.7)	1897 (39.9)	2689 (50.4)
Amniotomy	560 (19.3)	1518 (31.9)	2112 (39.6)
Oxytocin	172 (5.9)	603 (12.7)	981 (18.4)
Analgesia during labour			
Nitrous oxide	199 (6.9)	1565 (32.9)	2887 (54.2)
Epidural	224 (7.7)	901 (19.0)	1487 (27.9)
Narcotic	122 (4.2)	713 (15.0)	1877 (35.2)
Mode of delivery			
Spontaneous vaginal	2605 (89.9)	3910 (82.3)	4007 (75.2)
Assisted vaginal	86 (3.0)	344 (7.2)	736 (13.8)
Cesarean	208 (7.2)	498 (10.5)	588 (11.0)
Among nulliparous women	158/1215 (13.0)	453/2428 (18.7)	481/2204 (21.8)
Among multiparous women	50/1684 (3.0)	45/2324 (1.9)	107/3127 (3.4)
Primary indication for cesarean delivery			
Breech	34 (1.2)	0	0
Dystocia	79 (2.7)	253 (5.3)	288 (5.4)
Nonreassuring fetal heart rate	32 (1.1)	112 (2.4)	143 (2.7)
Repeat cesarean section	2 (0.1)	0	0
Malposition or malpresentation	39 (1.3)	89 (1.9)	78 (1.5)
Other	22 (0.8)	44 (0.9)	79 (1.5)
Episiotomy among vaginal deliveries	84/2691 (3.1)	289/4254 (6.8)	800/4743 (16.9)
**Maternal outcome**			
Prolapsed cord	2 (0.1)	6 (0.1)	9 (0.2)
Uterine rupture	0	0	2 (0.04)
Postpartum hemorrhage	110 (3.8)	285 (6.0)	357 (6.7)
Blood transfusion	2 (0.1)	10 (0.2)	15 (0.3)
Obstetric shock	1 (0.03)	1 (0.02)	1 (0.02)
Death	0	0	0
Manual removal of placenta	28 (1.0)	85 (1.8)	90 (1.7)
Uterine prolapse	1 (0.03)	1 (0.02)	2 (0.04)
Infection			
Pyrexia*	19 (0.7)	68 (1.4)	154 (2.9)
Urinary tract infection	0	1 (0.02)	5 (0.1)
Puerpural fever	1 (0.03)	4 (1.0)	7 (0.1)
Wound infection	0	11 (0.2)	16 (0.3)
Perineal tear			
None	1578 (54.4)	2189 (46.1)	2291 (43.0)
First- or second-degree tear	1262 (43.5)	2387 (50.2)	2836 (53.2)
Third- or fourth-degree tear	34 (1.2)	137 (2.9)	183 (3.4)
Degree of tear unknown	25 (0.9)	39 (0.8)	21 (0.4)
Cervical tear	2 (0.1)	5 (0.1)	4 (0.1)

*Temperature > 38°C.

whose mothers were in the physician-attended cohort.

The single most important reason for readmission of neonates to hospital in North America is hyperbilirubinemia.[22] Close to 40% of newborns with hyperbilirubinemia born in hospital can be identified and treated before discharge.[23] Therefore, the higher rate of admission (or readmission if a hospital birth) among newborns in the planned home-birth group than of readmission in the planned hospital-birth group may have been linked to the need for treatment of hyperbilirubinemia, which, among babies born in hospital, may require a longer stay in hospital rather than readmission.

Strengths and limitations

Our study has many strengths. Because both home and hospital births were attended by the same cohort of midwives, we were able to conduct a true comparison of planned place of birth unconfounded by type of caregiver. Our study adds to the body of large cohort studies of planned home births that have reported

Table 3: Association between maternal interventions and outcomes and planned place of birth among the 12 982 women in the study

Intervention/outcome	Relative risk (95% CI)	
	Planned home birth with midwife v. planned hospital birth with midwife	Planned home birth with midwife v. planned hospital birth with physician
Electronic fetal monitoring	0.32 (0.29–0.36)	0.17 (0.16–0.19)
Augmentation of labour	0.59 (0.55–0.69)	0.47 (0.44–0.51)
Narcotic analgesia, intramuscular or intravenous	0.27 (0.22–0.32)‡	0.12 (0.10–0.14)
Epidural analgesia	0.39 (0.33–0.46)‡	0.28 (0.24–0.32)
Assisted vaginal delivery	0.41 (0.33–0.52)	0.22 (0.18–0.27)
Cesarean delivery	0.76 (0.64–0.91)‡	0.65 (0.56–0.76)
Episiotomy*	0.49 (0.38–0.63)‡	0.19 (0.15–0.23)
Third- or fourth-degree perineal tear*	0.43 (0.29–0.63)‡	0.34 (0.24–0.49)
Postpartum hemorrhage	0.62 (0.49–0.77)	0.57 (0.45–0.70)
Infection†	0.39 (0.13–1.14)	0.26 (0.09–0.75)
Pyrexia	0.45 (0.29–0.76)	0.23 (0.14–0.37)

*Among women having a vaginal delivery.
†Urinary tract infection, wound infection or puerperal fever.
‡Adjusted for parity.

Table 4: Association between neonatal outcomes and planned place of birth among the 12 982 births in the study

Outcome	Relative risk (95% CI)	
	Planned home birth with midwife v. planned hospital birth with midwife	Planned home birth with midwife v. planned hospital birth with physician
Perinatal death	0.61 (0.06–5.88)	0.55 (0.06–5.25)
Apgar score < 7 at 1 min	0.76 (0.66–0.88)	0.74 (0.64–0.86)
Apgar score < 7 at 5 min	0.92 (0.58–1.47)	0.99 (0.61–1.61)
Meconium aspiration	0.83 (0.38–1.81)	0.45 (0.21–0.93)
Asphyxia at birth	0.79 (0.30–2.05)	0.70 (0.27–1.83)
Birth trauma*	0.26 (0.11–0.58)	0.33 (0.15–0.74)
Resuscitation at birth†	0.23 (0.14–0.37)	0.56 (0.32–0.96)
Birth weight < 2500 g	0.44 (0.25–0.78)	0.95 (0.56–1.78)
Seizures	0.61 (0.12–3.03)	0.66 (0.13–3.38)
Oxygen therapy > 24 h	0.37 (0.24–0.59)	0.38 (0.24–0.61)
Assisted ventilation > 24 h	1.02 (0.34–3.04)	0.68 (0.24–1.93)
Admission to hospital after home birth or readmission if hospital birth	1.09 (0.83–1.42)	1.39 (1.09–1.85)

Note: CI = confidence interval.
*Subdural or cerebral hemorrhage; fracture of clavicle, long bones or skull; facial nerve injury; Erb palsy; or unspecified birth trauma.
†Intermittent positive pressure via endotracheal tube or chest compression, or use of drugs for resuscitation.

on the relative safety of home versus hospital birth.[3–6,10] Complete ascertainment of outcomes of all home births in a large population and the ability to attribute outcomes to place of birth rather than type of caregiver should extend this literature. It should add confidence to the safety of home birth in a context such as ours in which registered midwives have a baccalaureate degree or equivalent and are an integral part of the health care system. Our findings do not extend to settings where midwives do not have extensive academic and clinical training.[24]

Our study has limitations. Aspects of care in the home environment that reduce the risk of obstetric interventions during labour are poorly understood.[25–27] We cannot exclude the possibility that differences in findings between the groups were attributable to unmeasured characteristics of the women who chose home birth. Although our study cohorts were closely matched on prognostic variables, we do not underestimate the degree of self-selection that takes place in a population of women choosing home birth. This self-selection may be an important component of risk management for home birth and in that context is a desirable facet of study design. Our data indicate that screening for eligibility by registered midwives can safely support a policy of choice of birth setting.

Our study is further limited by postpartum documentation of planned place of birth among midwifery clients. Bias introduced by misclassification of planned place of birth would not have changed our conclusions. In the worst-case scenario, if all perinatal deaths attributed to planned hospital birth in the midwifery comparison group had actually been planned home births, our perinatal death rate of 4 per 2882 live and stillbirths would have been 1.4 per 1000 in the home-birth group. The difference between midwifery groups would not have been significant, and the rate of perinatal death in the planned home-birth group would still have been very low.

Conclusion

Our study showed that planned home birth attended by a registered midwife was associated with very low and comparable rates of perinatal death and reduced rates of obstetric interventions and adverse maternal outcomes compared with planned hospital birth attended by a midwife or physician. Our population rate of less than 1 perinatal death per 1000 births may serve as a benchmark to other jurisdictions as they evaluate their home-birth programs.

This article has been peer reviewed.

Competing interests: None declared.

Contributors: Patricia Janssen participated in the conceptual design of the study, the analysis and interpretation of data and the drafting of the manuscript and wrote the final version. Lee Saxell, Lesley Page, Michael Klein and Shoo Lee participated in the conceptual design of the study, the interpretation of data and the drafting of the manuscript. Dr. Robert Liston participated in the interpretation of data and the drafting of the manuscript. All of the authors critically reviewed the article for important intellectual content and approved the final version submitted for publication.

Acknowledgement: We thank Kenny Der of the British Columbia Perinatal Health Program for providing the data for our study and for assisting with the data analysis.

Funding: This study was funded by the Canadian Institutes of Health Research after peer review. The study sponsor had no role in the design of the study, the collection, analysis or interpretation of data, the writing of the report or the decision to submit the article for publication. The authors' work was independent of the funders.

REFERENCES

1. Anderson RE, Murphy PA. Outcomes of 11,788 planned home births attended by certified nurse-midwives. A retrospective descriptive study. *J Nurse Midwifery* 1995;40:483-92.
2. Janssen PA, Holt V, Myers S. Licensed midwife-attended, out-of-hospital births in Washington state: Are they safe?. *Birth* 1994;21:141-8.
3. Janssen PA, Lee SK, Ryan EM, et al. Outcomes of planned home births versus planned hospital births after regulation of midwifery in British Columbia. *CMAJ* 2002;166:315-23.
4. Chamberlain G, Wraight A, Crowley P. Birth at home. *Pract Midwife* 1999;2:35-9.
5. Wiegers TA, Kierse MJ, van der Zee J, et al. Outcome of planned home and planned hosptial births in low risk pregnancies: prospective study in midwifery practices in the Netherlands. *BMJ* 1996;313:1309-13.
6. Ackermann-Liebrich U, Voegeli T, Gunter-Witt K, et al. Home versus hospital deliveries: follow up study of matched pairs for procedures and outcome. *BMJ* 1996;313:1313-8.
7. Van Alten D, Eskes M, Treffers P. Midwifery in the Netherlands. The Wormerveer study: selection, mode of delivery, perinatal mortality and infant morbidity. *Br J Obstet Gynaecol* 1989;96:656-62.
8. Lindgren H, Radestad I, Christensson K, et al. Outcome of planned home births compared to hospital births in Sweden between 1992 and 2004. A population-based register study. *Acta Obstet Gynecol Scand.* 2008; 87: 751-9. Available: www.informaworld.com/smpp/content~content=a794025460~db=all~order=pubdate (accessed 2009 June 26).
9. Woodcock HC, Read A, Moore D, et al. Planned homebirths in Western Australia 1981-1987. *Med J Aust* 1990;153:672-8.
10. Gulbransen G, Hilton J, McKay L, et al. Home birth in New Zealand 1973–93: incidence and mortality. *N Z Med J* 1997;110:87-9.
11. Pang JW, Heffelfinger J, Huang G, et al. Outcomes of planned home births in Washington State: 1989-1996. *Obstet Gynecol* 2002;100:253-9.
12. Mori R, Dougherty M, Whittle M. An estimation of intrapartum-related perinatal mortality rates for booked home births in England and Wales between 1994 and 2003. *BJOG* 2008;115:554-9.
13. Society of Obstetricians and Gynaecologists of Canada. Midwifery. *J Obstet Gynaecol Can* 2003;25:239.
14. American College of Obstetricians and Gynecologists. *ACOG statement on home birth*. Washington (DC): The College; 2009. Available: www.acog.org/from_home/publications/press_releases/nr02-06-08-2.cfm (accessed 2009 June 26).
15. Cresswell JL, Stephens E. *Home births*. London (UK): Royal College of Obstetrics and Gynaecologists and Royal College of Midwives; 2007. Available: www.rcog.org.uk/womens-health/clinical-guidance/home-births (accessed 2009 July 31).
16. College of Midwives of British Columbia. *Guideline for vaginal birth after one previous low segment caesarean section*. 2nd ed. Vancouver (BC): The College; 2003. Available: www.midwiferygroup.ca/downloads/vbac/CMBC%20VBAC%20guidline.pdf (accessed 2009 July 28).
17. Cahill A, Stamilio D, Adibo A, et al. Is vaginal birth after cesarean (VBAC) or elective repeat cesarean safer in women with a prior vaginal delivery? *Am J Obstet Gynecol* 2006;195:1143-7.
18. *British Columbia perinatal database registry annual report 2007*. Vancouver (BC): British Columbia Perinatal Health Program; 2007.
19. Hintze J. *PASS Power Analysis and Sample Size for Windows*. Kaysville (UT): NCSS; 2000.
20. *British Columbia perinatal database registry overview*. Vancouver (BC): British Columbia Perinatal Health Program; 2003.
21. Mantel N. Chi-square tests with one degree of freedom: extensions of the Mantel–Haenszel procedure. *J Am Stat Assoc* 1963;58:690-700.
22. Brown AK, Damus K, Kim MH, et al. Factors relating to readmission of term and near-term neonates in the first two weeks of life. Early Discharge Survey Group of the Health Professional Advisory Board of the Greater New York Chapter of the March of Dimes *J Perinat Med* 1999;27:263-75.
23. Sgro M, Campbell D, Shah V. Incidence and causes of severe neonatal hyperbilirubinemia in Canada. *Can Med Assoc J* 2006;175:587-90.
24. Cunningham JD. Experiences of Australian mothers who gave birth either at home, at a birth centre, or in hospital labour wards. *Soc Sci Med* 1993;36:475-83.
25. Ng M, Sinclair M. Women's experience of planned home birth: a phenomenological study. *RCM Midwives J* 2002;5:56-9.
26. Janssen PA, Carty E, Reime B. Satisfaction with planned place of birth among midwifery clients in British Columbia. *J Midwifery Womens Health* 2006;51:91-7.
27. Johnson KC, Daviss BA. Outcomes of planned home births with certified professional midwives: large prospective study in North America. *BMJ* 2005;330:1416.

Correspondence to: *Dr. Patricia A. Janssen, School of Population and Public Health, University of British Columbia, 5804 Fairview Cres., Vancouver BC V6T 1Z3; fax 604 806-8006; pjanssen@interchange.ubc.ca*
